# Ethosomal (−)-epigallocatechin-3-gallate as a novel approach to enhance antioxidant, anti-collagenase and anti-elastase effects

**DOI:** 10.3762/bjnano.13.41

**Published:** 2022-05-31

**Authors:** Çiğdem Yücel, Gökçe Şeker Karatoprak, Sena Yalçıntaş, Tuğba Eren Böncü

**Affiliations:** 1 Faculty of Pharmacy, Department of Pharmaceutical Technology, Erciyes University, 38280 Kayseri, Turkey; 2 Faculty of Pharmacy, Department of Pharmacognosy, Erciyes University, 38280 Kayseri, Turkey; 3 Erciyes University, Ziya Eren Drug Application and Research Center, Kayseri, Turkey

**Keywords:** antioxidant effect, antiaging effect, epigallocatechin gallate, ethosomal gel, ethosome

## Abstract

Controlled release systems containing natural compounds have been successfully applied in cosmetics as antiaging products to enhance the penetration of active compounds through the skin. In this study, we aimed to develop novel ethosomal formulations containing a potent antioxidant, epigallocatechin-3-gallate (EGCG), and to evaluate their potential for use in cosmetics by determining their antioxidant and antiaging effects. Ethosomes (ETHs) were prepared via mechanical dispersion and characterized in vitro in terms of particle size (PS), zeta potential (ZP), polydispersity index (PDI), encapsulation efficiency percentage (EE%), and in vitro release. The best ETH formulation was used to prepare the ethosome-based gel (ETHG) by using Carbopol 980 as a gelling agent at a ratio of 1:1 (v/v). The gel formulation was evaluated regarding organoleptic properties, pH values, and viscosity. Stability studies were conducted for three months and changes in characterization parameters and residual EGCG content of ETHs were examined. Besides, for ETHG, organoleptic properties, pH values (every two weeks), and viscosity (first and twelfth week) were determined for three months. The 3-(4,5-dimethyldiazol-2-yl)-2,5-diphenyltetrazolium bromide (MTT) assay was used to test the cytotoxicity of the formulations and different EGCG solutions on the L929 cell line. The cell permeation properties and inhibitory effects of ETHs and ETHGs on collagenase and elastase enzymes were investigated compared to those of the solution form. Within the scope of antioxidant activity studies, 2,2-diphenyl-1-picrylhydrazyl (DPPH•) and 2,2'-azino-bis(3-ethylbenzothiazoline-6-sulfonic acid) (ABTS+•) radical scavenging and β-carotene/linoleic acid co-oxidation inhibitory effects were carried out. The optimized EGCG-loaded ETHs (F3) were within the nanoscale range (238 ± 1.10 nm). The highest encapsulation efficiency and in vitro release values were 51.7 ± 1.15% and 50.8 ± 1.70%, respectively. The ETHG was successfully formulated with F3-coded ETHs and the cytotoxicity test revealed that the formulations and the EGCG solution at different concentrations were nontoxic. In terms of cell permeability, enzyme inhibition, and antioxidant activity, the ethosomal formulations yielded better results compared to the EGCG solution. It was observed that the formulations had a long-term effect due to the stability of EGCG. The findings of the study underline the potential of antioxidant and antiaging effects of the developed ethosomal formulations for use in the cosmetic field.

## Introduction

Skin aging is the result of biological changes, such as wrinkles, sagging, loss of elasticity, and thickening of the skin and it is caused by intrinsic (occur slowly and vary considerably between populations) and extrinsic factors. The main components of the connective tissue responsible for the elasticity and resistance of the skin in the dermis, (i.e., the middle layer of the skin) are collagen and elastin, and the changes in these two components play an important role in the skin aging process [1–2]. The production of reactive oxygen species (ROS) or free radicals through ultraviolet (UV) radiation, smoking, pollution, and normal endogenous metabolic processes triggers the skin aging process. Elastase and collagenase enzymes induced by the formation of ROS accelerate the aging process and cause loss of collagen and elastin fibrils. With the formation of free radicals, lipid peroxides and free oxygen radicals increase. Damage to phospholipids, which contain large amounts of unsaturated fatty acids and are sensitive to degradation by hydroxyl radicals, deteriorates the structure of the cell membrane [3].

Antioxidants are compounds that capture and stabilize free radicals and thus protect cells from oxidation-induced damage by slowing/inhibiting oxidation. They are of critical importance in maintaining the structural integrity of cells/tissues and ensuring the continuity of their functions, which include the ability to prevent side effects of free radicals [4–6].

Nowadays, natural products are preferred as the main treatment or complementary treatment of some diseases. Natural products can also be used as components in cosmetics, and the interest in these products has increased for this reason. Green tea (*Camellia sinensis*) is one of the most important natural products due to its safety and many benefits as a medicinal source. Epigallocatechin-3-gallate (EGCG), one of the major components of green tea, is a catechin-derived compound with a high antioxidant activity. It also has a protective effect against cardiovascular diseases, diabetes, cancer, and neurodegenerative disorders [7–9]. However, there are some difficulties in the formulation of EGCG such as the first pass metabolism effect, enzymatic degradation, and low bioavailability [8,10].

Skin delivery, besides being painless and noninvasive, has many advantages such as controlled drug delivery, reduced dose frequency, avoidance of first pass metabolism by the liver, and it can be self-administered. It includes the concept of topical drug delivery aimed at treating a local dermatological disorder without the need to target the systemic circulation by using transdermal drug delivery for systemic circulation [11–12]. Topical application of many antioxidants via cosmetics is one of several approaches to reduce oxidative damage in the skin. However, antioxidants are generally unstable and may degrade upon light exposure. Therefore, it is important to properly formulate antioxidants in order to overcome these problems and enhance their effectiveness [13–15]. Upon application of various topical formulations, lipophilic drugs can easily penetrate into the skin, while hydrophilic drugs, such as EGCG, are limited in terms of penetration. The skin displays selective permeability for the penetration of many drugs. The stratum corneum in the epidermis, which is the top layer of the skin consisting of three layers, acts as an important barrier against penetration into the skin [9,16]. To overcome penetration limitations, various techniques such as penetration enhancers, phonophoresis, electroporation, iontophoresis, needle-free injection, and microneedles are used [17]. As an alternative option, vesicular delivery systems are also effective systems to enhance the penetration of drugs through the skin. As an example, ethosomes (ETHs) are soft and malleable nanovesicles composed of phospholipids, water, and high amounts of ethanol that can carry both hydrophilic and lipophilic drug molecules. They are also highly deformable and reach deep skin layers [18–19]. ETHs are similar to the lipid bilayer composition of cells in the epidermis, due to the presence of phospholipids in their structure, and thus their interaction with skin cells is high. They are unique systems that can be easily shaped by the presence of ethanol in their structures, and their vesicle membranes become very flexible such that they can be transported through pores much smaller than their own diameters [20–21]. In addition, the synergistic effect of phospholipids and ethanol enables the ETHs and drug molecules to reach the deeper layers of the skin [19,22–23]. The skin penetration mechanism of drug-loaded ETHs is explained by both the ETH effect and the ethanol effect. While ethanol increases drug penetration by increasing the fluidity of intercellular lipids, ETHs can penetrate the deeper layers of the skin by interacting with the fluidized lipids of the loaded drug [20,24–26]. ETHs have proven to be effective delivery systems for the topical application of cosmetic products. Topically applied ETHs can increase the residence time of cosmetic active compounds in the stratum corneum and epidermis, allowing them to easily penetrate into the deeper layers of the skin. In addition, these systems have been proven to protect the skin from exogenous oxidants by preserving the structure of antioxidants with low stability and enhancing their effectiveness [13,15].

Permanence in the application area is important in topically applied formulations. Given their viscous structure, gel formulations can increase the residence time of a given substance on the skin and contribute to the penetration of drugs by moisturizing the skin as they contain sufficient water content in their structure [9].

In the literature, there is only one review study of nanocarriers in which ETHs, developed with natural compounds/plant extracts, are used in the field of cosmetics. In that study, information is given about the use of ETHs developed with different plant extracts other than EGCG [15].

In this current study, we aimed to develop ethosomal formulations for the use of EGCG in cosmetics due to its widely known strong antioxidant effect, which has been emphasized in many studies. In addition, we focused on enhancing the long-term antioxidant activity of the active compound with appropriate formulations and determining its inhibitory effects on elastase and collagenase enzymes, which accelerate the aging process in the skin, and our findings provide a novel and important contribution to the literature.

## Results and Discussion

### High-performance liquid chromatography assay

For the quantitative determination of EGCG, the optimal wavelength of 292 nm was used. The calibration curve obtained from high-performance liquid chromatography (HPLC) was linear between 15.625 to 1000 µg/mL with a high correlation coefficient (y = 1.0548x + 4.7737, *r*^2^ = 0.999). The peak of EGCG was detected at 5.93 min. The method was found to be sensitive and reproducible.

### Cytotoxicity

**Figure 1:**
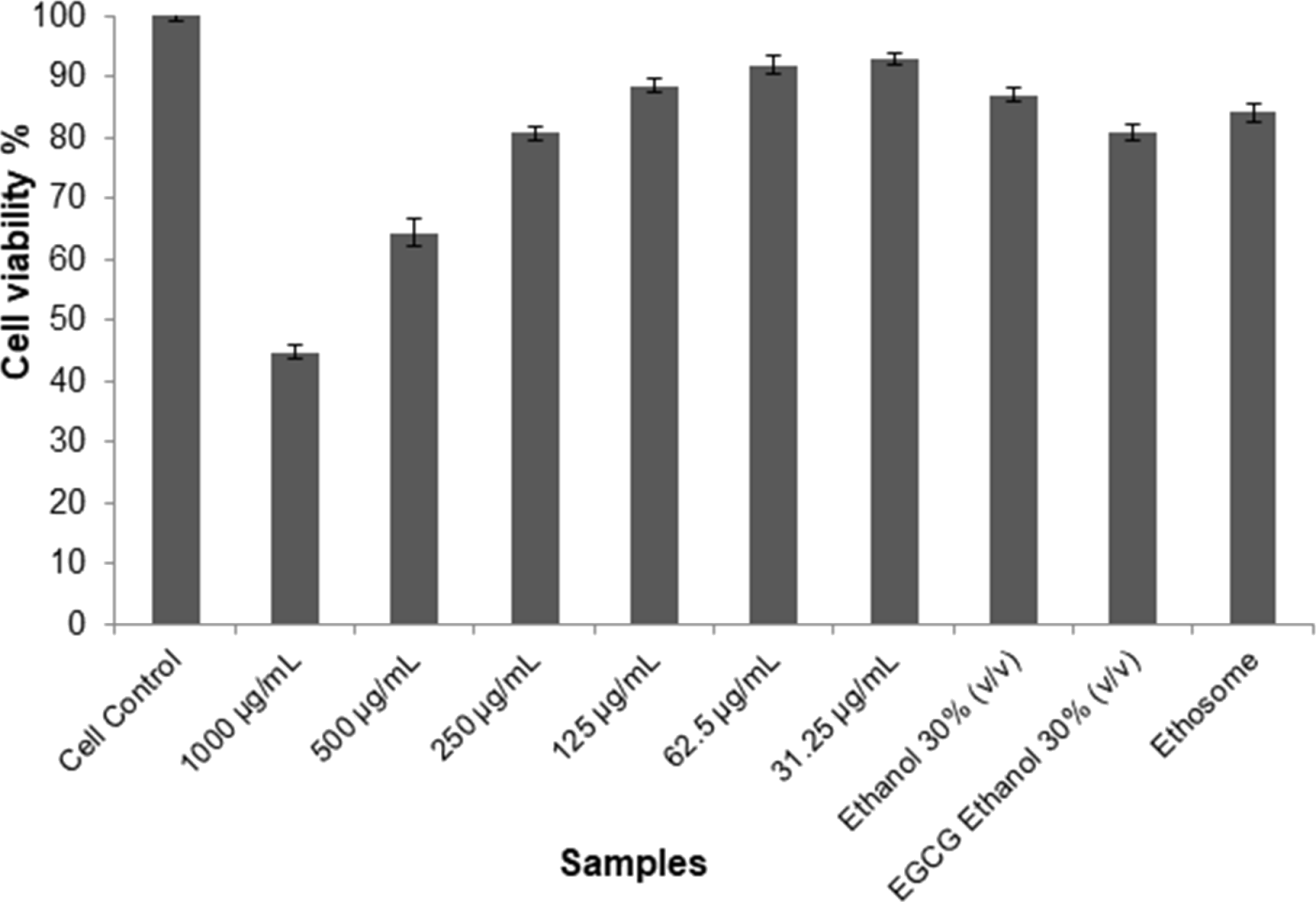
Percentage of cell viability of L929 cells incubated with samples for 24 h. (Values are expressed as mean ± standard deviation, *n* = 6).

L-929 cells are frequently used in cytotoxicity studies and shown as a reference cell line by international standards (ISO 10993 part 5, 1999; ISO 7405, 1997) for cytotoxicity tests [27], which are used for transdermal penetration studies and to determine cytotoxic concentrations of many samples. According to 3-(4,5-dimethyldiazol-2-yl)-2,5-diphenyltetrazolium bromide (MTT) tests, the cell viability was found to be 64.4% or higher for the hydroethanolic solution (30% v/v), ETH formulation, and different concentrations of EGCG. These results are given in Figure 1.

These obtained high viability rates demonstrate that all EGCG concentrations of 500 µg/mL and below, which is the initial concentration in the preparation of ETHs, did not cause any cellular toxicity. In addition, the hydroethanolic solution (30% v/v) and ethosomal formulations were not toxic to the cells and can be safely used. It has been stated in many studies that ETHs and other phospholipid-based carrier systems were not cytotoxic due to their similarity to the cell membrane structure. For our active substance EGCG, the dose determined based on cell viability results of 50% or more is appropriate [22,28].

In previous studies, the cytotoxic effect of EGCG, whose antioxidant effect has been proven many times, has been investigated, especially on cancer cell lines. Although there is no cytotoxicity study on the L929 cell line, one study investigated the anticancer activity of polymeric nanoparticles developed with many compounds (curcumin, EGCG, green tea extract, resveratrol, saponins, silymarin, and grape seed extract). Those nanoparticles target multiple signaling pathways and cause growth inhibitory effects on human cancer cells without causing toxicity problems in normal cells. In general, it has been confirmed that they are relatively nontoxic in healthy L929 cells but toxic in cancer cell lines [29].

### Preparation and characterization of formulations

Like many phenolic compounds, EGCG is not sufficiently effective due to its unstable structure and low solubility. In addition, toxicity may occur with an increasing dose [10]. Therefore, overcoming these problems with nanocarrier systems provides significant advantages to researchers. ETHs protect a given compound against environmental factors and also enhance penetration through the skin due to the ethanol in their composition [22]. In the current study, ethosomal formulations of EGCG with strong antioxidant properties were developed and their efficiencies were assessed and compared to the EGCG solution form. Six different ethosomal formulations were developed and characterized in vitro. Optimization of ETHs composed of 2–4% (w/v) of soya phosphatidylcholine (SPC) and 15–45% (v/v) of ethanol was performed based on characterization parameters (Table 1).

**Table 1:** Compositions and characterization parameters of six different EGCG-loaded ETHs^a^.

	F1	F2	**F3**	F4	F5	F6

ethanol (%) (v/v)	15	15	**30**	30	45	45
phospholipid (%) (w/v)	2	4	**2**	4	2	4
particle size (nm ± SD)	232 ± 1.09	242 ± 1.12	**238 ± 1.10**	243 ± 1.15	288 ± 2.01	294 ± 2.30
zeta potential (mV ± SD)	−29.3 ± 2.05	−28.2 ± 3.15	**−32.1 ± 1.14**	−30.4 ± 2.11	−30.1 ± 2.04	−32.3 ± 3.01
polydispersity index (PDI ± SD)	0.215 ± 0.005	0.252 ± 0.081	**0.210 ± 0.015**	0.232 ± 0.014	0.258 ± 0.014	0.274 ± 0.032
encapsulation efficiency (EE) (% ± SD)	43.2 ± 2.00	41.3 ± 2.01	**51.7 ± 1.15**	50.1 ± 1.15	51.4 ± 1.14	50.3 ± 2.01
in vitro release (% ± SD)	45.2 ± 1.23	48.4 ± 1.06	**50.8 ± 1.07**	49.5 ± 1.18	47.6 ± 1.87	47.9 ± 1.54

^a^Values are expressed as mean ± standard deviation, *n* = 3.

**Figure 2:**
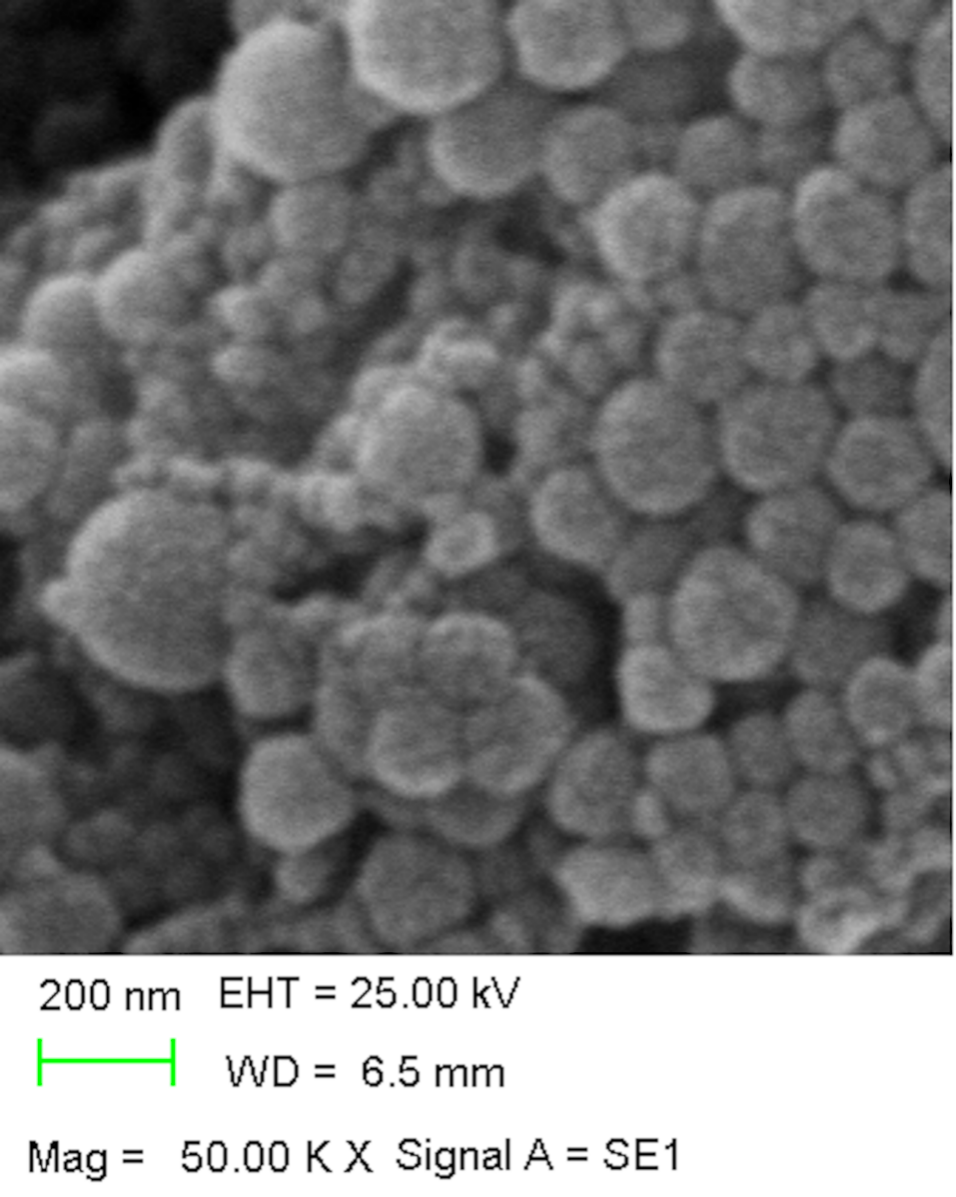
Scanning electron microscopy image of ETHs.

The F3 formulation was chosen as the optimum one. A sample of F3 was examined by scanning electron microscopy (SEM) (Figure 2).

As seen in Table 1, increasing particle size and particle size distribution were determined depending on the increasing lipid ratio and ethanol ratio. It is possible to say that ethanol gives a net negative charge to the ethosomal system and provides it with some degree of steric stabilization [30–31]. The negative zeta potential in all of our formulations is thought to be due to the presence of ethanol. Conversely, there is a decrease in the encapsulation efficiency due to the increasing lipid ratio. All formulations showed approximately 50% of in vitro release, but the highest rate was obtained in the F3-coded ETH.

Afterward, the ethosomal gel formulation was prepared by dispersing the F3 formulation in 1% (w/v) Carbopol 980 gel. Next, its organoleptic properties (i.e., color and homogeneity), viscosity and rheological properties, and pH values were evaluated. It was found suitable to use Carbopol 980 as the gelling agent at a ratio of 1:1 (v/v) due to its suitable organoleptic properties, pH value, viscosity, and its ability to homogeneously mix with the ethosome. A transparent, homogeneous, and smooth ethosomal gel system with a skin-compatible pH value (5.60) was obtained (Table 2).

**Table 2:** Evaluation of ethosomal gel properties^a^.

Formulation	Organoleptic properties	pH ± SD	Viscosity (cPs ± SD)

ethosomal gel	homogenous, transparent, smooth	5.60 ± 0.05	120 ± 0.80

^a^Values are expressed as mean ± standard deviation, *n* = 3.

El Kayal et al. conducted a study that provided the basis for the development of formulations as ETHs, transfersomes, and transethosomes to maximize the therapeutic efficacy of EGCG. The formulation compositions were optimized to produce vesicles with acceptable physical properties for these systems, and the effects of changes in the composition of the formulations on their characterization were examined. It was emphasized that appropriate optimization of vesicular systems is necessary to yield their therapeutic efficacy in topical applications [32]. In another study, an ethosomal formulation containing paroxetine hydrochloride for transdermal administration was developed and characterized in vitro. Different concentrations of lipid and ethanol were used during the preparation of formulations and their effects on the characterization parameters were interpreted. The optimal F2 formulation, selected from different ETHs exhibiting encapsulation efficiencies ranging from 40–64%, showed no significant changes in PS and polydispersity index (PDI) in a three-week stability study at room temperature. They also showed the highest in vitro permeability [33]. In order to increase the effectiveness of anthralin against psoriasis and reduce its side effects, various liposomal and ethosomal formulations were prepared with different compositions and characterized in terms of drug encapsulation efficiency, size, and morphology. The determined optima formulations were distributed on various gel bases and drug release kinetics were investigated. The formulations used had PSs in the nanoscale range and drug encapsulation efficiency values were over 97.2% and 77% [34]. In our study, both the ETHs and ETHG systems have been proven to be effective and safe in treatments with the cell permeation rate and effectiveness results obtained in our study, and this is also compatible with the literature.

### Stability studies

**Figure 3:**
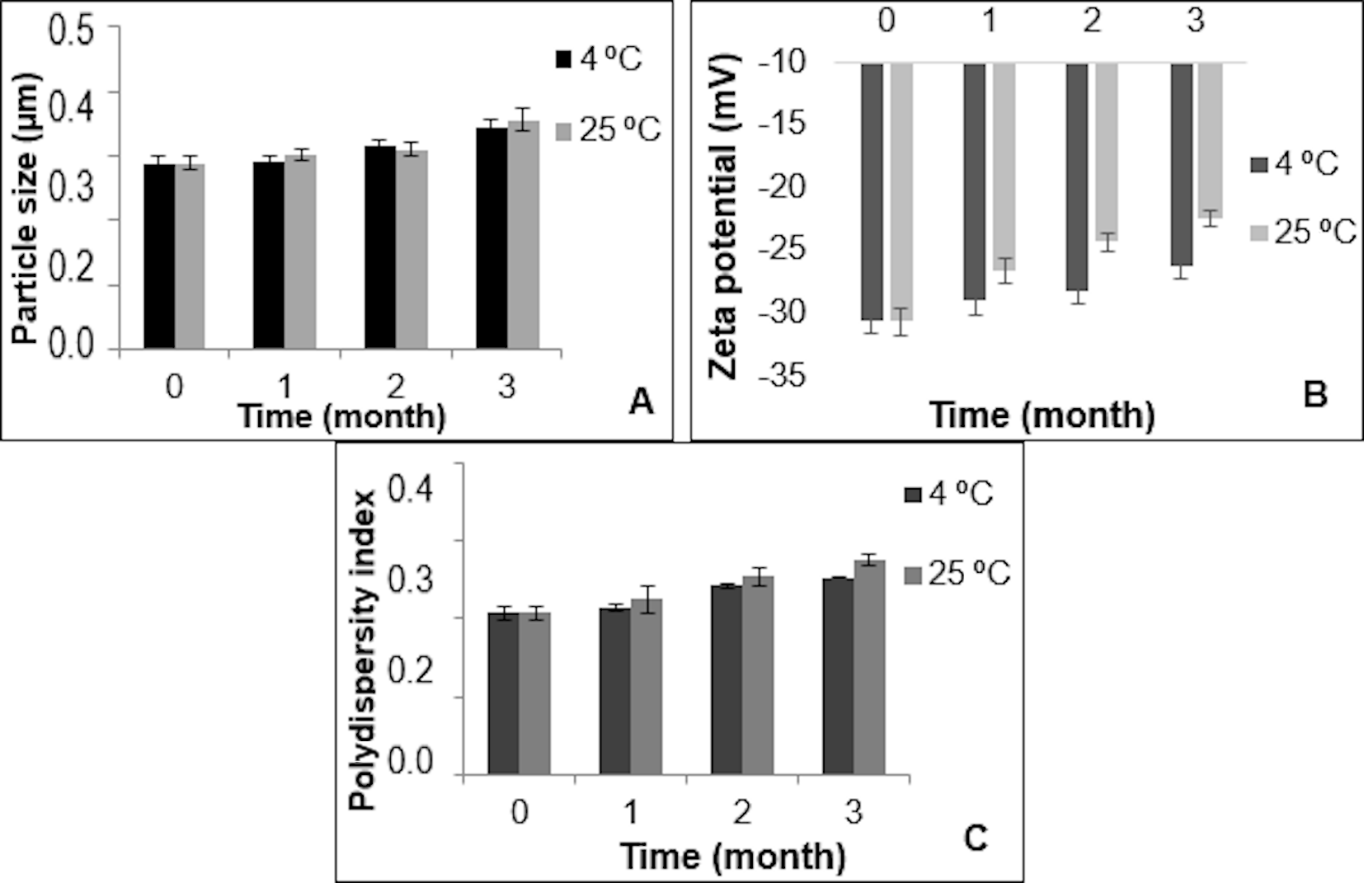
The changes in the PS, ZP, and PDI of the: (A, B and C) suspended ETHs at 4 and 25 °C. (Values are expressed as mean ± standard deviation, *n* = 3).

**Figure 4:**
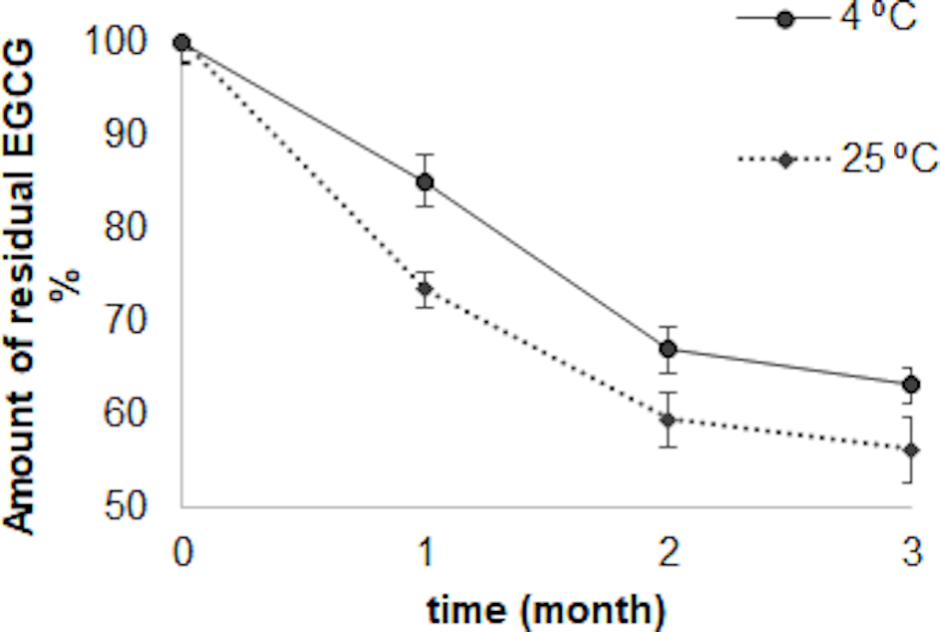
Degradation of EGCG in suspended ETHs at 4 and 25 °C (error bars represent standard deviations, *n* = 3).

Stability studies were performed at different conditions (4 and 25 °C + 60% relative humidity) for ETHs and ETHGs. PS and ZP values were periodically monitored once a month. The EGCG contents of ETHs were determined and degradation kinetics profiles were obtained (Figure 3 and Figure 4, respectively).

As seen in Figure 4, EGCG degradation was found to show first-order kinetics for ETHs. The degradation of EGCG in suspended ETHs was slower and lower at 4 °C compared to its degradation at 25 °C. No significant decrease in the drug content of the formulations was observed after three months at 4 °C (*p* > 0.005). In formulations stored at 25 °C, ± 60% relative humidity, both the amount of EGCG and the shelf life decreased with increasing temperature. Shelf lives of suspended ETHs were calculated as 25 days at 4 °C (correlation coefficient, *r*^2^ = 0.994) and 22 days at 25 °C (*r*^2^ = 0.991), respectively.

In ETHGs, it was determined that the gel was transparent at 4 °C, and transparent/yellowish at 25 °C, ± 60% relative humidity. The gel system showed good physicochemical properties at both 4 and 25 °C. The gel system did not show changes within the experimental error after three months. Although the pH value of the ethosomal gel slightly decreased over time with increasing temperature, it was still in the appropriate range for skin pH values (Table 3).

**Table 3:** Stability of ethosomal gels^a^.

Time (week)	pH ± SD 4 °C	pH ± SD 25 °C	Viscosity (cPs ± SD) 4 °C	Viscosity (cPs ± SD) 25 °C

0	5.60 ± 0.05	5.60 ± 0.05	120 ± 0.80	120 ± 0.80
2	5.59 ± 0.02	5.57 ± 0.04		
4	5.59 ± 0.08	5.53 ± 0.07		
6	5.57 ± 0.06	5.50 ± 0.04	115 ± 0.92	114 ± 1.00
8	5.54 ± 0.08	5.50 ± 0.06		
10	5.52 ± 0.09	5.49 ± 0.06		
12	5.51 ± 0.09	5.47 ± 0.08		

^a^Values are expressed as mean ± standard deviation, *n* = 3.

There are limited studies on EGCG-loaded ETHs. In a study conducted by Gwak et al., the effect on the stability of ETHs developed to prevent the degradation of EGCG under different storage conditions was observed. The stability values of the solution and the ethosomal formulation of EGCG upon exposure to UV or high temperature were compared, and it was noted that the degradation of EGCG under UV was delayed by the ETHs and by the incorporation of tocopherol as an antioxidant in the formulation [35]. In another study, cream-based formulations of different vesicular systems (liposomes, ethosomes, and transfersomes) containing *Curcuma longa* extract were prepared and these semisolid systems were subjected to stability tests by storing them at 4 °C for 3 months. These formulations were evaluated in terms of pH values, viscosity, smoothness, stickiness, and spreadability. According to the physicochemical evaluation of the cream formulations, their consistency, color, odor, spreadability, pH, and viscosity values did not show any change [36]. In a study by Shukla et al., the best formulation, which was determined among the ETHs containing different components, was dispersed in a Carbopol 980 gel matrix prepared at 0.5–1.0% and 1.5 w/w ratios. The gel formulation was evaluated in terms of pH values, viscosity, spreadability, in vitro release, washability, and extrudability. Stability studies were carried out with the optimized formulation which was stored at 4 °C, room temperature, and 40 °C for 45 days. It was emphasized that the ETHG prepared with 1% (w/w) Carbopol 980 exhibited zero-order release profile at a targeted site for a relatively longer period of time and better characteristics in the stability study, thus making it more pharmaceutically acceptable [37].

### Permeation studies

Permeation studies of EGCG from solution, ETHs, and ETHGs were performed and the amounts of EGCG at the end of 24 hours were found to be 39.1%, 40.3%, and 39.8%, respectively. The lower cell permeation percentages of EGCG compared to that of the in vitro release study can be explained by the limiting aspect of the cell monolayer.

ETHs are relatively new vesicular transporters consisting of phospholipids, ethanol, and water. The intriguing properties of ETHs are due to their high ethanol content. They promote targeted penetration through the stratum corneum and in deep skin layers, which are advantageous features compared to conventional vesicular systems such as liposomes, which have limited skin penetration and mostly remain in the upper layer of the stratum corneum. The release of the therapeutic agent occurs by the fusion of these vesicles with the cell membranes in the deeper layers of the skin [11]. ETHs have been reported to help many active substances to be kept in the skin for a longer period of time and penetrate into deeper layers. In a study by Fathalla et al., the gel-based formulations tested, especially with the ETH-based gel system, showed prolonged drug release in the treatment and yielded more successful results in the permeation study in rats in comparison to studies using liposomes. An improvement of 81.84% was achieved in the area affected by psoriasis [34]. Likewise, it can be said that better therapeutic results are obtained with ethosomal-based systems. Kaur and Saraf prepared and characterized different vesicular systems (liposomes, ETHs and transfersomes) containing *Curcuma longa* extract for a photoprotective effect in the skin damaged by UV radiation. Cream-based formulations were prepared with those systems containing high loading efficiency and low polydispersity. In that research, in which the vesicular systems were comparatively examined, it was once again proved that nanovesicles loaded with photoprotective plant extracts included in the creams were effective in penetrating through the skin, as a result of moisturizing lipid components, and it also showed a positive effect on skin hydration [36].

### Antioxidant activity

**Figure 5:**
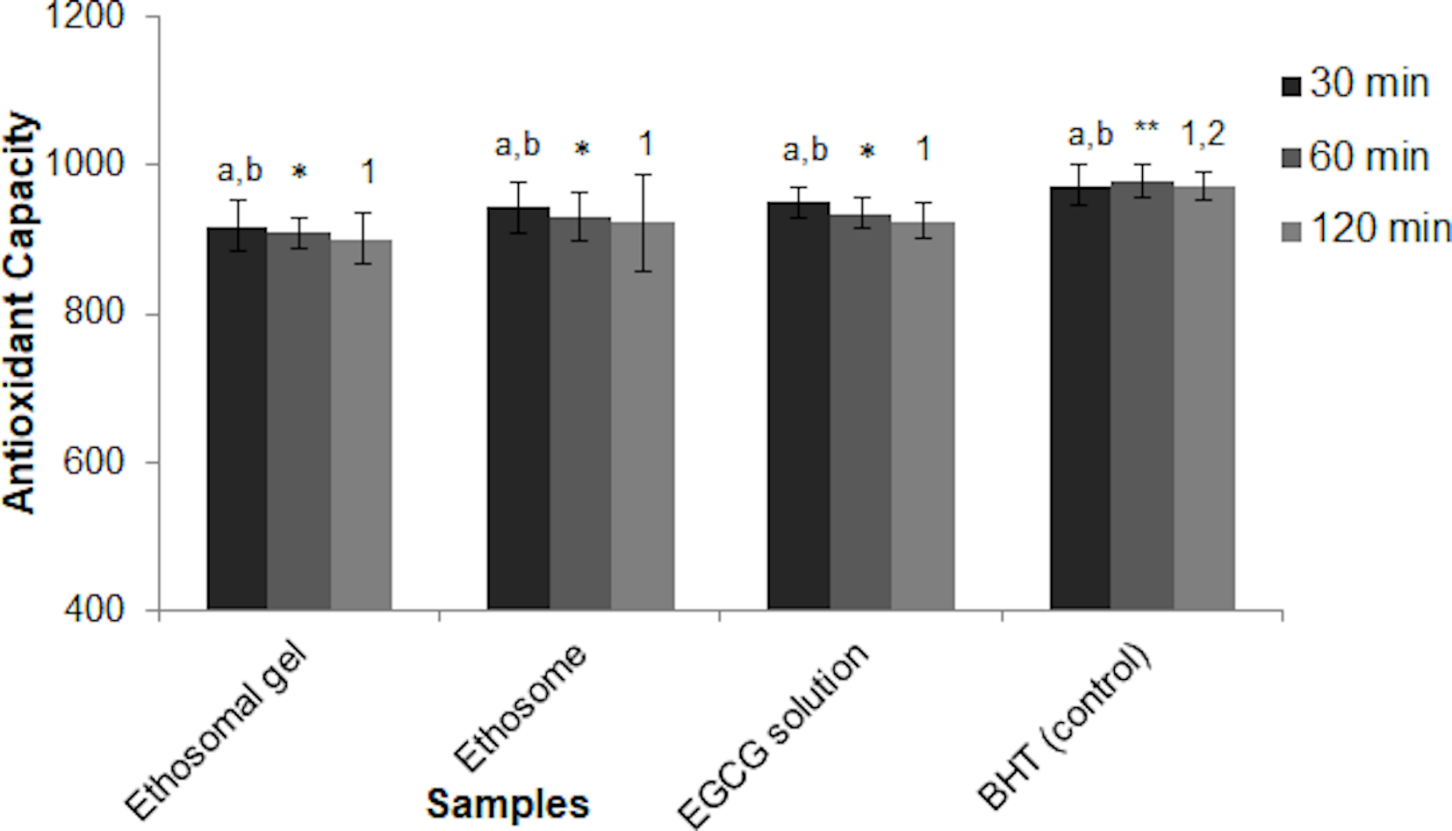
β-Carotene/linoleic acid co-oxidation inhibitory effects of EGCG in different samples [BHT: control group. Values are expressed as mean ± standard deviation, *n* = 3, bars with the same lowercase letter (a,b), (*,**), and (1,2) are not significantly different (*p* > 0.05)].

In the aging process, despite the long-term effects of oxidative damage on cells and tissues, one of the strategies to slow down or eliminate the harmful effects of aging is to maintain the balance between ROS production and antioxidant and cellular defense mechanisms. Along with the formation of ROS, pathological conditions such as cancer, heart diseases, and diabetes, as well as effects of sun damage, such as photoaging, wrinkling, crusting, dryness, and hyper pigmentation are seen [1]. In this scenario, defense with strong antioxidants is quite effective. The antioxidant activity of EGCG has been known for years and has been proven by many studies. It is stated in the literature that its antioxidant activity can be detected after ex vivo permeation studies [38]. We measured the antioxidant activity of the ethosomal formulations compared to that of an EGCG solution and our findings were given in Figure 5 and Table 4.

**Table 4:** The DPPH• and ABTS+• radical scavenging effects of samples^a^. The antioxidant capacity of the samples was determined via the Trolocks equivalent antioxidant capacity (TEAC) assay.

Samples	DPPH % inhibition ± SD	TEAC (mmol/L/Trolox) ± SD

EGCG solution	80.4 ± 1.50	2.05 ± 0.060
ETHs	85.2 ± 1.70	2.37 ± 0.046
ETHGs	84.7 ± 3.90	2.35 ± 0.089

^a^Values are expressed as mean ± standard deviation, *n* = 3.

High antioxidant effects were found at the end of the permeation study performed for 24 hours compared to those of the solution of EGCG, with which the ethosomal formulations were developed. The antioxidant effects of the solution form (500 µg/mL) were observed similarly in ethosomal formulations loaded (51.7%) and released (50.8%) at lower doses of EGCG (Table 4). This effect was thought to occur as a result of the long-term release of formulated EGCG at a lower dose while protecting it from environmental factors, and the success of the formulated EGCG was proven. In a previous study, the radical scavenging effect of 2,2-diphenyl-1-picrylhydrazyl (DPPH•) applied to ultra-deformable vesicular systems prepared with EGCG was determined. Ascorbic acid was used as the standard antioxidant compound and the antioxidant effect obtained by formulating EGCG showed better IC_50_ values compared to the those of the standard (14.2 µg/mL and 1.54 µg/mL, respectively) [32]. However, the β-carotene/linoleic acid co-oxidation inhibitory effects of our ethosomal formulations were different when compared to those of the synthetic antioxidant butylated hydroxytoluene (BHT) used as the control group (Figure 5). Here, it was thought that the very strong antioxidant capacity of BHT was dominant.

Studies of ethosomal formulations developed with EGCG are scarce. To the best of our knowledge, there is only one study that shows the optimization conditions to encapsulate green tea extract into ETHs. In this study they also examined in vitro and in vivo activities of EGCG, the main component of green tea, on rat skin when applied as a topical formulation. Two of the different ethosomal formulations of green tea extract which showed higher encapsulation efficiency percentage (EE%), lower PDI, as well as optimum release were determined as optima and were applied to rats in a gel base form. Total antioxidant capacity, catalase activity, malondialdehyde (MDA), and thiobarbituric acid reactive substance (TBARs) levels were determined from blood samples collected after oral and transdermal administration of the formulations to rats. It was stated that ETHs exhibited a controlled release rate, the levels of TBARs and MDA decreased in the groups supplemented with green tea extracts compared to those of the control group, and a greater decrease was observed in the transdermally administered groups compared to the orally administered groups. In vivo study data also highlighted the improved antioxidant effect of green tea extract with transdermal application. The results support our findings that the ethosomal formulations are successful in penetrating the lipid-rich biological membrane [39].

### Inhibition of collagenase and elastase enzymes

**Figure 6:**
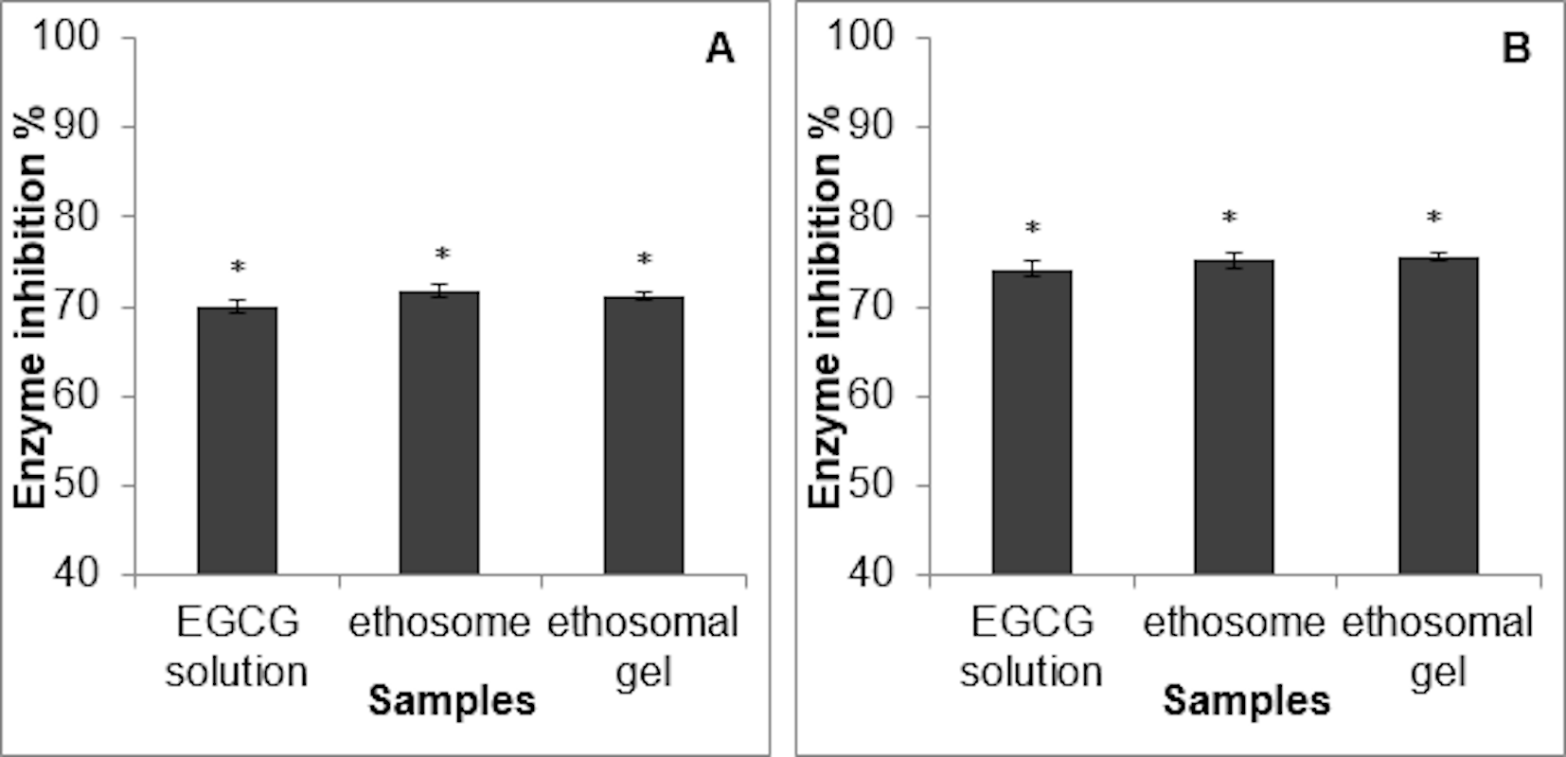
Inhibition values (%) of the: (A) collagenase and (B) elastase enzymes upon exposure to the EGCG solution, ETHs, and ETHGs. (Values are expressed as mean ± standard deviation, *n* = 3, significantly different groups *p < 0.05.).

At the end of the cell permeation studies, the inhibitory effects of the penetrated amount of EGCG on the collagenase and elastase enzymes were measured with kits. Collagenase enzyme inhibition values upon exposure to the EGCG solution, EGCG-loaded ETHs, and ETHG were found to be 68.0 ± 1.54%, 71.9 ± 1.23%, and 71.7 ± 1.52%, respectively. Elastase enzyme inhibition values upon exposure to the EGCG solution, EGCG-loaded ETHs, and ETHG were found to be 67.2 ± 1.60%, 75.2%, and 75.6%, respectively (Figure 6).

The inhibition of both enzymes upon exposure to the solution form of EGCG was found to be significantly lower with nanoformulations. It is thought that this low inhibition failed to maintain the efficacy of the solution form throughout the experiment, whereas sustained release formulations provided higher inhibition. Previous studies have proven that green tea is a functional herb that can be used as an antiaging agent in natural health products, including food, health, and cosmetic products [40]. EGCG is one of the major compounds of green tea, which is effective in anti-wrinkle treatments by inhibiting matrix metalloproteinase (MMP) enzymes. It is seen that studies on its antiaging effectiveness are mostly done using plant extracts containing EGCG. Although it has been shown in the literature that it is promising to formulate EGCG by loading it into vesicular conduction systems for effective skin penetration, the nanoformulation and effect of EGCG used as an active substance is quite limited [8–9]. In one study, a nanotransfersome carrier system containing EGCG and hyaluronic acid, which can penetrate into the lower layers of the skin given its elastic structure such as ETHs, was developed and its effect against skin aging caused by UV radiation was investigated. The inhibitory effect on MMP-2 and MMP-9 enzymes, which are responsible for the degradation of type IV and type VII collagen, respectively, was measured in HaCaT cells, and a higher inhibition of MMP enzymes was achieved with higher penetration against unformulated EGCG [8]. In a previous work from our team, an ethosomal formulation developed with rosmarinic acid showed higher inhibition on collagenase and elastase enzymes compared to that of solution and liposome forms. Data supporting the access of ETHs to the deeper layers of the skin were obtained [22].

## Conclusion

In conclusion, ETHs were studied as a possible vehicle for antioxidant and antiaging effects of EGCG via topical administration. EGCG-loaded ETHG was successfully prepared based on the optimum formulation. The findings of the study confirmed that ETHs are very promising carriers compared to the solution form of EGCG for the transdermal delivery of EGCG. The parameters considered were antioxidant and enzymes inhibition effects, transport properties across cell monolayers, and better stability profiles. These evident effects of EGCG via ethosomal formulations might help to optimize the targeting of dermal sites for the study of antiaging effects. These formulations can also serve as a useful tool to prolong the contact time of EGCG with the skin. Thus, new opportunities may be created for well-controlled and modern topical application of EGCG in cosmetology.

## Experimental

### Materials

(−)-Epigallocatechin-3-gallate, 3-(4,5-dimethyldiazol-2-yl)-2,5-diphenyltetrazolium bromide (MTT), SPC, trypsin-EDTA solution, dimethyl sulfoxide (DMSO) for cell culture, and penicillin/streptomycin solution were purchased from Sigma, USA. Carbopol 980 was purchased from the Abdi İbrahim Pharmaceutical, Industry and Trade Company, Turkey. All the other chemicals used were of analytical grade. The NTCT clone 929 cell line (L929, connective mouse tissue) was acquired from the American Type Culture Collection (ATCC^®^ CCL-1™), Manassas, USA. Eagle's Minimum Essential Medium (EMEM) (ATCC^®^ 30-2003™) was purchased from Biochrom, Germany. Cell culture flasks and plates were purchased from Corning^®^. Cedex. Smart Slides and Trypan Blue solution were purchased from Roche (Switzerland). Mouse collagenase and elastase enzyme ELISA kits were obtained from Sunredbio, Shanghai.

### HPLC assay

The quantitative determination (i.e., the amount of EGCG permeated in the receptor compartment during in vitro release and cell permeation experiments) of EGCG was carried out by HPLC assay on an Agilent 1200 Series system using acetic acid 1%. An acetonitrile (1.5:8.5 v/v) mixture was used as the mobile phase delivered at a flow rate of 1 mL/min. A ten-microliter injection was eluted in a C18 column (150 × 4.6 mm, 5 μm) at room temperature. The maximum spectrum between 200–400 nm was determined with a UV spectrophotometer (Shimadzu 1800). The column eluent was monitored at an optimal wavelength [41–42]. For the standard curve, working solutions of EGCG ranging from 15.625 to 1000 µg/mL, prepared from the stock solution, were used.

### Cytotoxicity

The L929 mouse fibroblast cells were grown in EMEM (ATCC^®^ 30-2003) supplemented with 10% fetal bovine serum and 1% penicillin/streptomycin in an incubator at 37 °C under a 5% CO_2_ atmosphere. For cytotoxicity experiments, the MTT colorimetric assay was used [24]. Suspensions of L929 cells (100 μL, 2.5 × 10^4^ cells/well) were seeded onto 96-well culture plates which were kept at 37 °C for 24 h for cell adhesion [43]. The cells were treated with different EGCG solutions (31.25–1000 µg/mL) and EGCG-loaded ETHs and incubated for 24 h. After removing the culture medium, 100 µL of fresh medium containing 13 µL of MTT solution (5 mg/mL in PBS) was added to each well and the plate was incubated at 37 °C for 4 h. The content of each well was removed and insoluble purple formazan crystals were then dissolved in 100 μL of DMSO. After shaking, the color density was measured at 570 nm with a multiwell ELISA reader (Biotech Synergy HT, USA). The control group viability was considered as 100% and the results were presented as percentages.

### Formulation studies

#### Preparation of formulations

The ethosomal system was prepared using mechanical dispersion [22,24]. The prepared ETHs consisted of 2–4% (w/v) phospholipids, 15–45% (v/v) ethanol and the effects of phospholipid and ethanol concentration on the characterization properties of the formulations were investigated. The SPC was added to the round-bottomed flask, dissolved with chloroform: methanol (1:3 v/v), and evaporated to form a thin lipid film on the wall of the flask in a rotary evaporator (Heidolph) under vacuum. For hydration of the dry film, the EGCG hydroethanolic solution (15%, 30%, and 45% v/v) was used. The preparation was vortexed for 15 min followed by sonication using an ultrasonic bath (Wise Clean, Korea, 210 W, 40 kHz), at 40% sonication strength, and three cycles of 30 min to obtain monodisperse vesicles and to enhance encapsulation efficiency [44–45]. Then, ETHs were collected by centrifugation at 10.000 rpm for 30 min.

For the preparation of ETHGs, initially 1.0% (w/v) of Carbopol 980 was added to distilled water, adjusted to pH 5.5 with triethanolamine in 1:1 (v/v) ratio, and allowed to swell overnight at room temperature. Then, the optimized ethosomal dispersion was gelled by mixing 1.0% (w/v) Carbopol 980 solution in the ratio of 1:1 (v/v).

#### Characterization of formulations

In the characterization studies, PS, ZP, and PDI of the developed ETHs were measured using a Zetasizer Nano ZS-Malvern. The ETHs were visualized using SEM. The content of EGCG entrapped in ETHs (EE%) was estimated after removal of the uncaptured drug determined by HPLC from the supernatant phase after ultracentrifugation. The encapsulation efficiency of the ETHs was calculated according to the following equation.


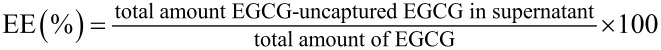


Furthermore, an in vitro release study for six ETHs was performed for 24 h at 37 °C using Franz diffusion cells with a 12.000 Dalton pore size dialysis membrane which was mounted between the donor and receptor compartments. Six different EGCG-loaded ETH suspensions (1 mL) were placed in the donor compartment. The receptor compartment contained 2.5 mL of phosphate buffer (pH 7.4) and was constantly stirred by a magnetic stirrer. The sink condition was maintained throughout the experiment and the temperature was maintained at 37 °C. Samples were withdrawn from the receptor compartment at the end of 24 h and analyzed by HPLC as described above.

The ETHG was characterized according to its organoleptic properties (color, homogeneity), and pH values (Mettler Toledo Seven Compact, Switzerland). In addition, viscosity and rheological properties were measured using the Brookfield viscometer (Brookfield, USA) with a spindle no. 14 at 100 rpm and 25 °C.

#### Stability studies

In the stability studies, the vesicular ETH suspensions were stored in amber-colored glass bottles under various conditions (4 and 25 °C, ± 60% relative humidity) and the changes in the PS, ZP, PDI, and residual EGCG content of the formulations were reviewed over a 90-day period. In ETHGs, the stability was evaluated in terms of organoleptic properties and pH values every two weeks [9]. Viscosity and rheological properties were measured using the Brookfield viscometer at the first and twelfth week.

### Cell permeation studies

Cell permeation studies were conducted after L929 cells were seeded at 2.5 × 10^5^ cells/well [24] onto 6-well Transwell^®^ plates with a pore diameter of 0.4 µm. The ethosomal formulations (1 mL) were placed in the donor compartment. The amounts of EGCG permeated through L929 cell monolayers at the end of 24 h in parallel with the in vitro release study were calculated by HPLC.

### Antioxidant activity

Within the scope of antioxidant activity studies, DPPH• and 2,2'-azino-bis(3-ethylbenzothiazoline-6-sulfonic acid) (ABTS+•) radical scavenging effects taken at the end of the cell permeation study were determined based on methods described by Gyamfi et al. and Re et al., respectively [46–47]. In addition, β-carotene/linoleic acid co-oxidation inhibitory effects were determined according to the β-carotene bleaching method of Velioğlu et al. [48–49].

### Inhibition of collagenase and elastase enzymes

At the end of the cell permeation studies performed with the EGCG solution and its ethosomal formulations, the inhibitory effects of EGCG obtained from the receptor compartment on collagenase and elastase enzymes, induced by the formation of free radicals, were measured by using collagenase and elastase ELISA kits.

### Statistical analysis

All data in this study were considered as means ± SD, and one-way ANOVA was used for statistical analysis. The GraphPad InStat software, version two, was used.
